# Platelet mitochondrial DNA methylation: a potential new marker of cardiovascular disease

**DOI:** 10.1186/s13148-015-0078-0

**Published:** 2015-04-16

**Authors:** Andrea A Baccarelli, Hyang-Min Byun

**Affiliations:** Laboratory of Environmental Epigenetics, Exposure Epidemiology and Risk Program, Department of Environmental Health, Harvard T.H. Chan School of Public Health, 677 Huntington Ave., Boston, MA 02115 USA; Human Nutrition Research Centre, Institute of Cellular Medicine, Newcastle University, Biomedical Research Building, Campus for Ageing and Vitality, Newcastle upon Tyne, NE4 5PL UK

**Keywords:** Mitochondrial epigenetics, DNA methylation, Platelet, Cardiovascular disease

## Abstract

**Background:**

Platelets are critical in the etiology of cardiovascular disease (CVD), and the mitochondria in these cells serve as an energy source for platelet function. Epigenetic factors, especially DNA methylation, have been employed as markers of CVD. Unlike nuclear DNA methylation, mitochondrial DNA (mtDNA) methylation has not been widely studied, in part, due to debate about its existence and role. In this study, we examined platelet mtDNA methylation in relation to CVD.

**Results:**

We measured mtDNA methylation in platelets by bisulfite-PCR pyrosequencing and examined associations of CVD with methylation in mitochondrial genes; cytochrome c oxidase (*MT-CO1*, *MT-CO2*, and *MT-CO3*); tRNA leucine 1 (*MT-TL1*); ATP synthase (*MT-ATP6* and *MT-ATP8*); and NADH dehydrogenase (*MT-MD5*). We report that CVD patients have significantly higher mtDNA methylation than healthy controls in *MT-CO1* (18.53%, *P* < 0.0001), *MT-CO2* (3.33%, *P* = 0.0001), *MT-CO3* (0.92%, *P* < 0.0001), and *MT-TL1* (1.67%, *P* = 0.0001), which are involved in ATP synthesis. Platelet mtDNA methylation was not related with age, BMI, and race in this study.

**Conclusions:**

Our results suggest that platelet mtDNA methylation, which could serve as non-invasive and easy-to-obtain markers, may be implicated in the etiology of CVD.

**Electronic supplementary material:**

The online version of this article (doi:10.1186/s13148-015-0078-0) contains supplementary material, which is available to authorized users.

## Background

Cardiovascular disease (CVD) is the leading cause of death in the United States, accounting for more than 800,000 deaths every year, corresponding to one of every three deaths, or one every 40 s [1]. As the proportion of Americans aged over 75 years is expected to increase from 6% in 2000 to 11% in 2050, the health-care burden due to CVD is similarly predicted to rise dramatically in the coming years [2].

Platelets play an important role in CVD, both in the pathogenesis of atherosclerosis and in the development of acute thrombotic events. Abnormal platelets, either quantitatively or qualitatively, are associated with CVD [3-6]. Platelets process a rapid and complex signaling cascade that regulates adhesion, aggregation, and activation to mediate thrombus formation. Platelets have a greater rate of ATP turnover than resting mammalian muscle that contains high levels of mitochondria [7], suggesting an essential role for mitochondria in platelet function, and energy demand escalates during platelet activation and secretion [8,9]. Mitochondrial DNA (mtDNA), as with nuclear DNA, can be methylated by machinery existing inside of the mitochondria [10-12] and can mediate the control of mitochondrial gene expression [13]. Platelets lack a nucleus, and therefore, the mitochondrial genome is the only genetic material in these cells. Therefore, the epigenetic regulation of mitochondrial genes in platelets is critical to understanding their implication in the development of CVD.

The mitochondria contain their own DNA molecules, which are copies of a genome of approximately 17 kb in circular form. The mitochondrial genome contains 37 genes, 13 of which are protein encoding while 24 encode tRNAs and rRNAs. There are several aspects of the mitochondrial genome that distinguish it from the nuclear genome. Firstly, the mitochondrial genome lacks histone complexes and retrotransposons (such as LINE-1 and *Alu*) [11]. Secondly, there are many copies of nuclear mitochondrial DNAs (‘NUMTs’), which are derived from cytoplasmic mtDNA that has been incorporated into the nuclear genome [14]. Thirdly, since the level of mtDNA methylation is far less than that of nuclear DNA, the existence and function of mtDNA methylation has been debated. However, there is growing evidence that mtDNA methylation has been overlooked. Vanyushin BF *et al*. first reported DNA methylation and DNA methyltransferase (DNMT) activity in the mitochondria in 1971 [15]. Shock *et al*. reported mitochondrial DNMT1 activity, mtDNA methylation, and control of gene expression through this pathway [16]. Byun *et al*. demonstrated a relationship between mtDNA methylation and environmental air pollution exposure - an established CVD risk factor - in healthy humans [10]. Indeed, mitochondrial DNA methylation has been proposed as a next-generation biomarker and diagnostic tool [17]. As every cell type contains differing numbers of mitochondria and different levels of their activity, it is expected that mtDNA epigenetic patterns will differ by tissue and cell type. Therefore, studying mitochondrial epigenetic patterns by single cell type is critical to understand the effects of mtDNA methylation on disease.

DNA methylation markers in the nuclear genome of unfractionated peripheral blood lymphocytes (PBL) associated with CVD risk have produced inconsistent results [18-21] and have been particularly challenging to develop. The possible reason for this difficulty is that PBLs are a mixture of multiple leukocyte subtypes [22], and therefore the observed changes in molecular markers in at-risk individuals, which may differ in each of the cellular subtypes, may simply reflect differences in the proportions of circulating cellular subtypes [23]. Furthermore, each leukocyte subtype has a variable risk-predictive relationship with CVD [24]. Therefore, investigating platelet mitochondria provides a unique opportunity to solve the biological riddle that arises with the use of a mixed population of blood cells (PBLs/buffy coat). In addition to this, studying a key blood cell type for CVD development may provide greater insight into developing biomarkers for CVD risk. Platelet mitochondria can be obtained non-invasively and can be easily isolated from both fresh and frozen plasma samples, thus they provide an easy-to-handle biospecimen for clinical and preventive applications. However, the efficacy of using mtDNA as a biomarker has been controversial [17,25,26], mainly due to the sensitivity and detection limitation associated with low levels of mtDNA methylation. Therefore, it is critical to apply a sensitive technique to measure mtDNA methylation levels, such as by bisulfite pyrosequencing [10], in order to understand the effect of this epigenetic phenomenon in disease.

In this study, we measured and analyzed platelet mitochondrial DNA methylation levels of genes associated with ATP synthesis: three of four protein-encoding cytochrome c oxidase genes associated with the electron transport chain complex (*MT-CO1*, *MT-CO2*, and *MT-CO3*); ATP synthase (*MT-ATP6* and *MT-APT8*); and NADH dehydrogenase (*MT-ND5*). Additionally, we analyzed the tRNA leucine 1 gene (*MT-TL1*) that is known to have clinical significance [27-29] in CVD patients in comparison to healthy individuals. We utilized bisulfite pyrosequencing, which is a sensitive and well-established DNA methylation detection method. We observed that individuals with CVD showed higher methylation levels than healthy individuals in several mitochondrial genes in platelets.

## Results

### Characteristics of healthy individuals and CVD patients

We collected samples from 17 healthy individuals and 10 patients with CVD for the study. Due to failed pyrosequencing data points (see ‘Methods’ section), the number of healthy individuals and CVD patients that are compared for each gene differ slightly. Fifty-three percent of the healthy individuals are males (9/17) in comparison to 30% of CVD patients (3/10). The mean ages of the healthy individuals and CVD patients were 43.8 and 62.4 years, respectively. Our study contained a mix of races. Among the healthy individuals, 35% were Caucasian, 24% Hispanic, and 41% African-American. Among the CVD patients, 40% were Caucasian, 20% Hispanic, 30% African-American, and 10% Asian. The mean BMI was 30.4 for healthy individuals and 27.5 for the CVD patients. All ten CVD patients had hypertension, and the group also included two patients with atherosclerosis, one patient with atrial fibrillation, and three patients who were clinically diagnosed with CVD (Table 1). Patients were selected upon the basis of diagnosis with hypertension and/or atherosclerosis, as these are the most common causes of CVD.

**Table 1:** **Details of the plasma samples from healthy individuals and CVD patients**

**Sample ID**	**Gender**	**Age**	**Race**	**BMI**	**Diagnosis**	**Medications**
23	Male	46	Caucasian	25	-	-
24	Male	71	Hispanic	24	-	-
25	Male	54	Caucasian	34	-	-
27	Male	50	African-American	30	-	-
28	Male	38	African-American	34	-	-
29	Male	23	African-American	23	-	-
30	Male	22	Hispanic	24	-	-
31	Male	59	Caucasian	28	-	-
32	Male	52	African-American	32	-	-
33	Female	52	Caucasian	34	-	-
34	Female	59	Caucasian	31	-	-
35	Female	44	Hispanic	26	-	-
36	Female	41	Hispanic	30	-	-
37	Female	19	African-American	54	-	-
38	Female	26	African-American	27	-	-
39	Female	48	Caucasian	32	-	-
41	Female	41	African-American	28	-	-
08	Male	62	Asian	25	Hypertension, high cholesterol, benign prostatic hyperplasia	Flomax, Hyzaar, Zocor, Urocit, Vitamin D
09	Male	66	African-American	25	Hypertension, hypertensive cardiovascular disease (HCVD)	Naprosyn, Flomax, Lisinopril, Toprol XL, Norvasc, ASA, Lipitor, Aciphex
16	Male	64	Caucasian	26	Coronary artery disease, hypertension, lung cancer	ASA, Carvediolol, Dexamethasone, Lovaza 1gram, Ramipril, Vytorin
10	Female	54	Hispanic	31	Atherosclerosis, hypertension	Synthroid, tenoretic
11	Female	83	Caucasian	24	Atrial fibrillation, hypertension	Coumadin 5 mg, Cardura
12	Female	44	Hispanic	26	Atherosclerosis, hypertension, chest pain	Aspirin, Atenolol, Diovan HCT
13	Female	65	Caucasian	36	Hypertension, lung cancer, secondary mal neoplasms	Keppra, Lisinopril Hydrochlorothyazide, Metoprolol tartrate, Pravachol, Sucralfate, Tarceva, Tarceva, Xanax
14	Female	55	African-American	31	Hypertension, end stage of kidney disease	Cozaar, Renvella, Lasetalol
15	Female	59	Caucasian	27	Hypertension, high cholesterol, type 1 diabetes	Actonel, Advair, Benicar, Novolin, Caltrate, Spiriva, Synthroid, Tocor
17	Female	72	African-American	26	Hypertension, heart disease, osteoarthritis, type 2 diabetes	Endocet, Lantus, Ferrous Fumerate, Colace, Aspirin, Simvastatin, Amlodipine, Hydralazine, Lisinopril, Januvia

### Platelet mtDNA methylation in healthy individuals and CVD patients

**Figure 1:**
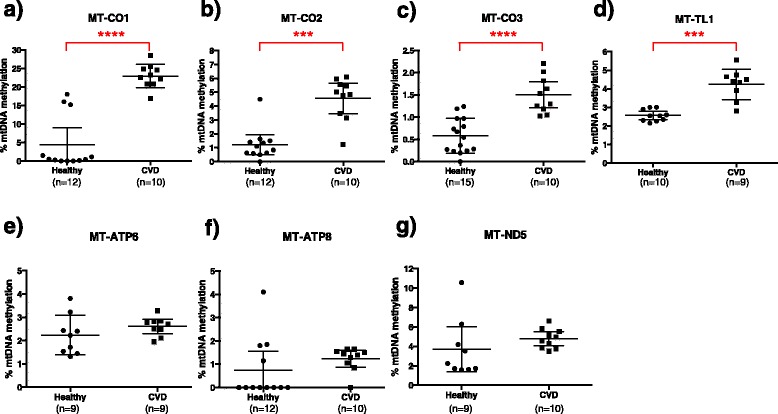
Distribution of percentage mtDNA methylation in healthy individuals and CVD patients. Distribution of percentage mtDNA methylation in healthy individuals and CVD patients for **(a)** *MT-CO1*, **(b)** *MT-CO2*, **(c)** *MT-CO3*, **(d)** *MT-TL1*, **(e)** *MT-ATP6*, **(f)** *MT-ATP8*, and **(g)** *MT-ND5* are shown. The values from all individuals are displayed, with lines corresponding to the mean and 95% confidence interval values. ****P < 0.0001;***P = 0.0001.

We measured platelet mtDNA methylation in healthy individuals and patients with CVD by bisulfite PCR combined with pyrosequencing. Mean *MT-CO1* DNA methylation was 4.45% (min = 0 and max = 18.04, SD: 7.27) in healthy individuals and 22.97% (min = 16.87, max = 28.49, SD: 3.17) in CVD patients. The difference in mean mtDNA methylation levels between healthy individuals and CVD patients was 18.53% (*P* value <0.0001, 95% CI: 13.25 to 24.20, by Mann–Whitney U test) (Figure 1a). Mean *MT-CO2* DNA methylation was 1.22% (min = 0 and max = 4.50, SD: 1.14) in healthy individuals and 4.55% (min = 1.23 and max = 6.11, SD: 1.52) in individuals with CVD. The difference in mean mtDNA methylation levels between healthy and CVD individuals was 3.33% (*P* value = 0.0001, 95% CI: 2.13 to 4.71) (Figure 1b). Mean *MT-CO3* DNA methylation was 0.58% (min = 0 and max = 1.24, SD: 0.39) in healthy individuals and 1.50% (min = 1.03 and max = 2.21, SD: 0.39) in individuals with CVD. The difference in mean mtDNA methylation levels between healthy and CVD individuals was 0.92% (*P* value <0.0001, 95% CI: 0.56 to 1.30) (Figure 1c). Mean *MT-TL1* DNA methylation levels were 2.57% (min = 2.15 and max = 3.00, SD: 0.31) in healthy individuals and 4.24% (min = 2.80 and max = 5.55, SD: 0.82) in individuals with CVD. The difference in mean mtDNA methylation levels between healthy and CVD individuals was 1.67% (*P* value = 0.0001, 95% CI: 1.00 to 2.25) (Figure 1d). We did not observe significant differences in mtDNA methylation for *MT-ATP6*, *MT-ATP8*, or *MT-ND5* (Figure 1e,f,g). We examined the association between mtDNA methylation at each site and the age, BMI, race, and gender of individuals, but none of these factors showed a significant association (Additional file 1).

### The predictive probability of platelet mtDNA methylation as a CVD biomarker

**Figure 2:**
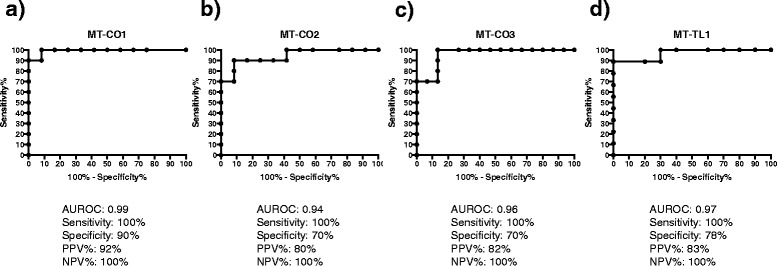
The predictive probability of platelet mtDNA methylation as a CVD biomarker. Nonparametric receiver operating characteristic (ROC) curves are shown for **(a)** *MT-CO1*, **(b)** *MT-CO2*, **(c)** *MT-CO3*, and **(d)** *MT-TL1.* Areas under ROC (AUROC) curves with sensitivity, specificity, and positive (PPV) and negative (NPV) predictive values are below the graph.

To determine the predictive probability of platelet mtDNA methylation as a CVD biomarker, we performed receiver operating characteristic (ROC) curve analysis. Areas under ROC curves (AUROCs) were estimated and compared by chi-square test. The AUROC was 0.99 for *MT-CO1* gene, 0.94 for *MT-CO2*, 0.96 for *MT-CO3*, and 0.97 for *MT-TL1.* The sensitivity and specificity were 100% and 90% for *MT-CO1* gene, 100% and 70% for *MT-CO2*, 100% and 70% for *MT-CO3*, and 100% and 78% for *MT-TL1* (Figure 2)*.*

### Purity determination of extracted mtDNA

**Figure 3:**
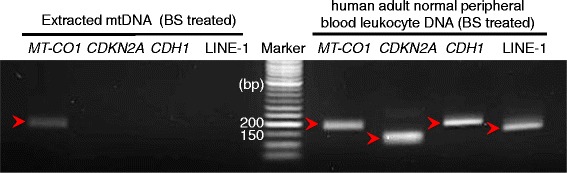
A representative gel following PCR amplification of mitochondrial and nuclear DNA sequences. Amplification of *MT-CO1*, *CDH1*, *CDKN2A*, and LINE-1 in extracted mtDNA (lanes 1 to 4) and control DNA from human adult normal peripheral blood leukocyte (Biochain, Newark, CA, USA) (lanes 6 to 9) were visualized on a 2% agarose gel. The fifth lane contains a DNA size marker.

In order to examine the extent of nuclear DNA contamination, particularly from NUMTs, in extracted platelet mtDNA prior to pyrosequencing performance, we performed PCR amplification of a gene located in the mitochondrial genome (*MT-CO1*), two single-copy genes located in the nuclear genome (*CDH1* and *CDKN2A*) and a repetitive element (LINE-1) for which multiple copies exist in the nuclear genome. In addition to extracted mtDNA, human adult normal peripheral blood leukocyte DNA (Biochain, Newark, CA, USA) was used as a control. The PCR was performed using bisulfite-converted DNA, as it was for the performance of pyrosequencing. We observed amplification of mtDNA (*MT-CO1*) in both bisulfite-treated platelet mtDNA and leukocyte DNA. However, we did not observe PCR amplification of the nuclear DNA-encoded *CDH1*, *CDKN2A*, or LINE-1 in the extracted platelet mtDNA, demonstrating absence of nuclear DNA in these samples. A representative gel is shown in Figure 3.

### Technical replicates and quantitative validation of pyrosequencing assays

**Figure 4:**
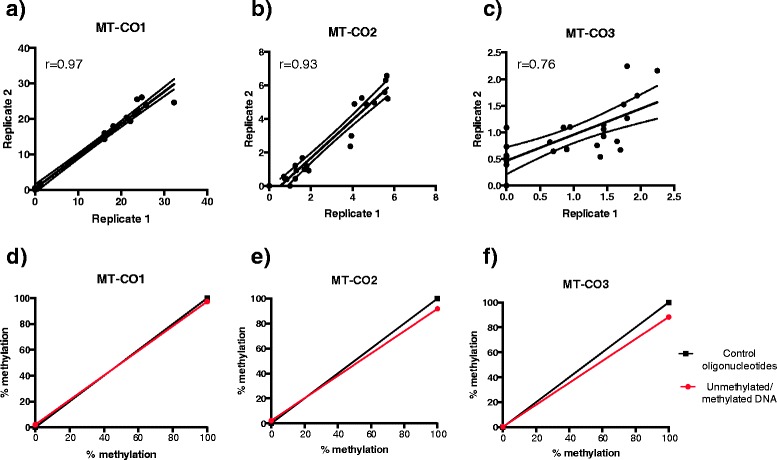
Technical replicates and quantitative validation of mtDNA pyrosequencing assays. Spearman correlation coefficients (*r*) of the technical replicates (replicates 1 and 2) for the designed pyrosequencing assays were **(a)** 0.97 for *MT-CO1*, **(b)** 0.93 for *MT-CO2*, and **(c)** 0.76 for *MT-CO3*. The plot curve is displayed with a 95% confidence band. The quantitative validation of 0% and 100% methylated DNA for the **(d)** *MT-CO1*, **(e)** *MT-CO2*, and **(f)** *MT-CO3* assays (red circle) were compared with 0% and 100% control oligonucleotides (black rectangle).

We performed technical replicates to assess the precision of mtDNA methylation determination, as well as to confirm the observed small but significant differences in mtDNA methylation for three selected assays: *MT-CO1*, *MT-CO2*, and *MT-CO3*. The Spearman rank correlation coefficient between technical replicates for the *MT-CO1* assay (methylation range: 0.00% to 32.30%) was 0.97 (*P* value <0.0001, 95% CI: 0.92 to 0.99) (Figure 4a). For the *MT-CO2* assay (methylation range: 0.00% to 6.31%), the correlation was 0.93 (*P* value <0.0001, 95% CI: 0.82 to 0.97) (Figure 4b), and for *MT-CO3* (methylation range: 0.00% to 2.25%), it was 0.76 (*P* value <0.0001, 95% CI: 0.51 to 0.89) (Figure 4c). In order to quantitatively validate our newly designed mtDNA pyrosequencing assays, we used universal whole-genome unmethylated and methylated DNA to serve as 0% and 100% methylation controls, respectively. The measured methylation values from amplification of these cell line-based controls was compared to 0% and 100% methylated control oligonucleotides, generated by synthesis of sequences with the presence of a T or C nucleotide at the interrogated CpG sites to represent 0% and 100% methylated DNA, respectively. The *MT-CO1* assay reported 2.2% methylation in the cell line-extracted unmethylated control and 97.3% in the methylated control (Figure 4d), the *MT-CO2* assay reported 2.3% methylation in the unmethylated control and 91.8% in the methylated control (Figure 4e), and the *MT-CO3* assay reported 0.4% in the unmethylated control and 88.3% in the methylated control (Figure 4f).

## Discussion

In this study, we hypothesized that DNA methylation in platelet mitochondria is a potential contributor to CVD development through modulation of platelet activity. We examined platelet mtDNA methylation among healthy individuals and CVD patients, and we observed significantly higher mtDNA methylation of four genes in CVD patients. The predictive probability analysis demonstrates the potential utility in using platelet mtDNA methylation levels as a CVD biomarker. The role of mtDNA methylation in platelet function has yet to be fully elucidated, but our data is the first to characterize platelet mtDNA methylation as being implicated in CVD.

Due to tissue-differential and cell type-differential methylation patterns, using a mixture of multiple cell types, as routinely found in human tissues, has long been a critical hurdle in clinical studies [22,23]. Peripheral blood DNA - which is no easier to isolate than platelet DNA - is derived from diverse types of leukocytes. Similarly, vascular tissues, such as heart tissues and blood vessels, are comprised of multiple cell types. The main cell types found in heart tissues include endothelial cells, epicardium cells, smooth muscle cells, cardiomyocytes, and fibroblasts [30], while several others, such as macrophages or white and red blood cells can infiltrate the tissues. As CVD patients exhibit changes in the counts and proportions of individual leukocyte subtypes, as well as in the numbers of different cell types in the heart and blood vessels, differences in DNA methylation observed in these composite tissue types that are associated with CVD (and other diseases) have been suggested to result solely from differences in cell type composition rather than from *bona fide* epigenetic changes. Even in the presence of *bona fide* DNA methylation changes independent of cell type compositions, analysis of DNA isolated from whole tissue will inherently provide results that are more difficult to interpret, as they cannot be traced to one or more of the specific cell types within the tissue. Our study has the unique advantage of being based on platelets, which is the only cell type in the human body that can be readily isolated without labor-intensive and costly procedures.

The mitochondrial genome is known to retain a very low level of DNA methylation, and we observed low levels of platelet mtDNA methylation in healthy individuals, with higher mtDNA methylation in those with CVD. The observed changes were relatively small in magnitude, except for *MT-CO1*, yet distinct between healthy and CVD individuals. What we observed in *MT-CO1* is an astounding difference (18.53%) in platelet mtDNA methylation from CVD patients. Previous studies have reported no difference in nuclear DNA methylation in blood samples collected after ischemic heart disease and/or stroke diagnosis compared with baseline [19], while Movassagh *et al.* reported comparatively small 2% to 5% differences in nuclear DNA methylation in left ventricular tissue from four cardiomyopathy patients [31]. Taken together, these findings demonstrate the utility of studying mtDNA methylation in platelets. Furthermore, platelet mtDNA methylation patterns showed little variation between individuals, which is perhaps the product of the study of a single cell type, but nonetheless were markedly different by CVD status. Compared to measured mtDNA methylation patterns using DNA from buffy coats [10], methylation levels were more homogenous in purified platelets. Further work is required to establish whether the magnitude of the observed changes in mtDNA methylation is sufficient to alter transcription of mitochondrially encoded genes or affect protein activity.

Platelets are involved in blood clot formation, and abnormal clotting is implicated in heart attacks, stroke, and other CVD risks. Platelet dysfunction is also related to activation and hyperaggregation, which are further CVD risk factors [32]. The mitochondria play a critical role in maintaining the normal function of the heart, and therefore mitochondrial dysfunction is related to the pathogenesis and development of various types of heart disease [33]. We observed a significant increase in mtDNA methylation of all three MT-CO subunits in platelets from CVD patients. COX is a component of the respiratory electron transport chain, with subunits 1 to 3 (*MT-CO1*, *MT-CO2*, and *MT-CO3*) forming the functional core of complex IV. Genetic mutations in COX genes are related to fatal metabolic disorders [34] which predominantly affect tissues with high energy demands, such as the heart. Further, lower platelet COX activity is related to sepsis mortality [35] that commonly occurs with cardiac dysfunction. Dysfunctional COX complexes are therefore known to be the most severe among the many classified mitochondrial diseases [34]. However, further work is required regarding platelet COX activity and quantity with regard to CVD.

Overweight and obese individuals have a high risk of developing CVD [36]. We did not observe an association between BMI and platelet mtDNA methylation in this study. However, higher mtDNA methylation levels, similar to those in CVD cases, were observed in three healthy individuals for *MT-CO1* (BMI: 26, 28, and 34, which correspond to being overweight and obese) and one individual for *MT-CO2* (BMI: 28, overweight) (Figure 1). It has not been possible to obtain follow-up data regarding whether these apparently healthy individuals developed CVD after the samples were taken. Whether platelet mtDNA can be a marker of CVD predisposition among obese and overweight individuals is therefore not yet clear.

We obtained platelet pellets by centrifugation and DNase I treatment to remove nuclear DNA on account of the possibility of contamination of the samples with NUMTs [37]. We did not observe any nuclear DNA in our samples of extracted platelet mtDNA, which could potentially have arisen from cell-free nuclear DNA or from other blood cells. However, while nuclear DNA could not be amplified by PCR in the extracted mtDNA, we cannot exclude the possibility that a minor portion of nuclear DNA contamination could still exist in our extracted mtDNA. Therefore, caution is still required with the application of other techniques for the study of the mitochondrial genome, such as whole-genome sequencing or bisulfite sequencing.

We have chosen to use bisulfite pyrosequencing to measure locus-specific mtDNA methylation. As extensively proven, and also through our own experience, pyrosequencing is the most reliable, sensitive, and robust technique for studying DNA methylation. Therefore, we opted to use this method instead of other available high-throughput and genome-wide techniques, such as whole-genome bisulfite mtDNA sequencing,

There are several limitations to our study. We obtained plasma samples from donors, but the sample size is relatively small in comparison to other clinical studies. Nonetheless, we demonstrated both the feasibility of this approach and report definitive results in the data. Most importantly, this method is innovative and could prove to be beneficial to other relevant studies. As our study is a proof of principle, it can pave the way for larger-scale studies concerning the function of platelet mtDNA methylation in CVD development. While the CVD patients that we studied have multiple diagnoses, it is common to observe comorbidity of CVD with other disease types, and therefore it was inevitable to include individuals with complex diagnoses.

Our study is also limited by the possibility of contamination with cell-free mitochondria in the platelet pellets that were isolated. Cell-free mitochondria are highly unlikely to be pelleted with platelets on account of the centrifugation speed that was used (1,400 × *g*), as low-speed centrifugation separates platelets from the smaller and lighter particles, such as exosomes, microparticles, and cell-free mitochondria that are pelleted at over 12,000 × *g* [38]. Nonetheless, the possibility of trace quantities of cell-free mitochondria in the pellet cannot be excluded. We therefore sought to optimize our method to obtain platelet-enriched mtDNA by using DNase I treatment to remove cell-free mtDNA, in addition to the use of low-speed centrifugation to separate platelets from cell-free mitochondria. We examined the centrifuged platelets with transmission electron microscopy and confirmed that majority of cells, if not all, were platelets (data now shown due to a discrepancy of donor samples used for the study). It has been reported that some of the cell-free mitochondria present in plasma originate from platelets themselves [39], and so trace contamination with platelet-derived mitochondria and microparticles that contain mtDNA should not affect measured methylation levels, on account of the cells of origin being the same. A further limitation of this study is that pyrosequencing analyses failed in some of the samples due to an insufficient yield of mtDNA. We have purposely not applied a method using platelet-rich plasma in order for our findings to have greater significance through more direct implications for studies with blood samples collected by commonly used methods, such as those taken in clinical routine blood drawing. However, it is indeed possible to increase mtDNA yield and thereby increase the range of techniques and approaches that can be utilized when platelet-rich plasma is available.

## Conclusions

Our study is the first to characterize the possible role of platelet mitochondrial DNA methylation in relation to CVD risk. Focusing on a single, pure cell population that is critical to CVD etiology provides ease of interpretation for DNA methylation patterns in relation to disease. As platelets can be inexpensively isolated from stored plasma, our study demonstrates a novel approach that can be applied to many existing studies and which may yield risk and diagnostic biomarkers for CVD.

## Methods

### Human plasma

We purchased 17 fresh frozen human plasma samples from healthy individuals and 10 from individuals with CVD (BioreclamationIVT, Baltimore, MD, USA). Information about gender, ethnicity, age, diagnosis, and treatment were available from the vendor (Table 1).

### Extraction and bisulfite DNA conversion of mtDNA in platelets

We centrifuged 400 μl of plasma samples at 1,400 × *g* for 15 min at room temperature to obtain platelet pellets. To minimize contamination with nuclear DNA [37,40], we added 3 μl of DNase I (Roche Holding AG, BASEL, Switzerland, 10 U/μl), 3 μl incubation buffer (10×, 400 mM Tris–HCl, 100 mM NaCl, 60 mM MgCl_2_, 10 mM CaCl_2_; pH 7.9), and 3 μl water and incubated the samples for 3 h at 37°C. After incubation, we followed the protocol provided with the EZ DNA Methylation-Direct™ Kit (Zymo Research Corp., Orange, CA, USA) for extraction and sequential bisulfite-conversion of mtDNA. Briefly, we treated the samples with Proteinase K for 1 h at 50°C and centrifuged the samples at 10,000 × *g* to remove cell debris, with the supernatant retained. The standard bisulfite conversion protocol provided by the kit manufacturers was followed, with elution of mtDNA in 10 μl of elution buffer.

### Quantitative validation of pyrosequencing assay

We purchased universal methylated and unmethylated DNA samples (Zymo Research Corp., Orange, CA, USA) as 0% and 100% methylation controls. We used control oligonucleotides for 100% DNA methylation (PSQ-C oligo: 5′-TTGCGATACGACGGGAACAAACGTTG-3′) and 0% DNA methylation (PSQ-T oligo: 5′-TTGCGATACAACGGGAACAAACGTTG-3′). The sequencing primer for the control oligonucleotide is: 5′-AACGTTTGTTCCCGT-3′. The PSQ-C oligonucleotide (or PSQ-T oligonucleotide) and sequencing oligonucleotide were mixed in annealing buffer. Pyrosequencing was performed with a sequencing entry of C/TGTAT [41].

### PCR and pyrosequencing

We designed nine assays to interrogate mtDNA methylation based upon the GeneBank: J01415.2 (L-strand) mitochondrial genome sequence using the Meth Primer program [42]. The PCR and pyrosequencing primers are provided in the Additional file 1. We measured three CpGs sites for the *MT-ATP6* assay and two CpGs for all the other assays. We used 1 μl of bisulfite-treated mtDNA for PCR reactions with 10 μl GoTaq Green Master mix (Promega, Madison, WI, USA) and 10 μl of PCR products for pyrosequencing using the PSQ Q96 MD pyrosequencing system (QIAGEN, Valencia, CA, USA), as previously described. Briefly, 10 μl of PCR products for each sequencing reaction were immobilized on to streptavidin-coated beads (Streptavidin Sepharose HP, GE Healthcare Biosciences, Pittsburgh, PA, USA) in binding buffer (10 mM Tris–HCl, 2 M NaCl, 1 mM EDTA, 0.1% Rweeb 20; pH 7.6) for 10 min. The biotin-labeled template was purified using the pyrosequencing vacuum prep tool (QIAGEN, Valencia, CA, USA) and incubated with 10 pmol/reaction sequencing primer in annealing buffer (20 mM Tris-acetate, 2 mM MgAc_2_; pH 7.6). The DNA strands were denatured at 80°C for 2 min and reannealed at room temperature for 10 min. Sequencing was performed according to manufacturer’s instructions. The allele frequencies (% cytosine or % thymidine) were calculated from the peak heights, as analyzed by the allele quantification module in the PSQ 96 MD software (QIAGEN, Valencia, CA, USA). Percentage methylation was determined by the ratio of cytosine-to-thymidine conversion (methylation = % cytosine/(% cytosine + % thymidine)). We have excluded results from failed samples according to pyrosequencing built-in quality controls.

### Statistical analysis

We performed non-parametric Mann–Whitney tests to analyze the differences in mtDNA methylation between healthy and CVD patients. To analyze technical duplications of pyrosequencing performance, we computed Spearman’s rank correlation (*r*) as the data was not normally distributed. ROC curves were generated for each mtDNA assay. Maximum mtDNA methylation levels from healthy individuals for each marker were used as cutoffs to determine the sensitivity, specificity, PPV, and NPV. All graphs and statistical analysis were made using the Prism software (GraphPad software, La Jolla, CA, USA).

## Additional file

Additional file 1:
**Supplementary information 1, 2, and 3.** Supplementary information 1. Primer sequence and information. Supplementary information 2. Platelet mtDNA methylation level with age, BMI, and race. Supplementary information 3. Scatter plots of mtDNA methylation from healthy individuals and CVD patients by age, BMI, and race.
