# Supplementary material for: Platelet mitochondrial DNA methylation: a potential new marker of cardiovascular disease
Source: Clin Epigenetics. 2015 Apr 16;7(1):44. doi: 10.1186/s13148-015-0078-0 (PMC4404685; doi:10.1186/s13148-015-0078-0)

Title Page

Platelet mitochondrial DNA methylation: a potential new marker of cardiovascular disease

Andrea A. Baccarelli[1] and Hyang-Min Byun[1*†]

Supplementary Information

Supplementary Information 1. Primer sequence and information
Supplementary Information 2. Platelet mtDNA methylation level with age, BMI, and race
Supplementary Information 3. Scatter plots of mtDNA methylation from healthy individuals and CVD patients by age, BMI, and race

Supplementary Information 1. Primer sequence and information

Assay	Name	Sequence	Genomic Location	Annealing Temp. (ºC)
MT-CO1	Forward	TATTAATTGGTTTTTTAGGGTTTAT	(GeneBank: J01415.2) 6736-6760	52
	Reverse (bio)	CAACAAATCATTTCATATTACTTCC	(GeneBank: J01415.2) 6888-6912	
	Seq primer	TATTTATAGTAGGAAT	(GeneBank: J01415.2) 6778-6793	
	Seq entry	AGAC/tGTAGATATAC/tGAGTATAT	(GeneBank: J01415.2) 6797, 6807	
MT-CO2	Forward	TTTATGAGTTGTTTTTATATTAGGTTTAAA	(GeneBank: J01415.2) 8068-8097	52
	Reverse (bio)	ACTCCACAAATTTCAAAACATTAAC	(GeneBank: J01415.2) 8166-8190	
	Seq primer	TAAAAATAGATGTAAT	(GeneBank: J01415.2) 8094-8109	
	Seq entry	TTTC/tGGAC/tGTTTAAATTA	(GeneBank: J01415.2) 8113, 8117	
MT-CO3	Forward	TATATTATTTGTTTAAAAAGGTTTT	(GeneBank: J01415.2) 9419-9443	52
	Reverse (bio)	AATAAAAAACTCAAAAAAATCCTAC	(GeneBank: J01415.2) 9489-9513	
	Seq primer	TATATTATTTGTTTAAAAAGGTTTT	(GeneBank: J01415.2) 9419-9443	
	Seq entry	C/tGATAC/tGGGATAATTT	(GeneBank: J01415.2) 9444, 9449	
MT-ATP6	Forward	TTATAAATTTAGTTATGGTTATTTTTTTAT	(GeneBank: J01415.2) 8822-8851	52
	Reverse (bio)	AAACTAAAACATTTTTAATCTTAAAAC	(GeneBank: J01415.2) 8879-8905	
	Seq primer	TTATAAATTTAGTTATGGTTATTTTTTTAT	(GeneBank: J01415.2) 8822-8851	
	Seq entry	GAGC/tGGGC/tGTAGTGATTATAGGTTTTC/TGT	(GeneBank: J01415.2) 8855, 8861 (8862: G/A SNP)	
MT-ATP8	Forward	AAATTATAATAAATTTTGAGAATTAAAATG	(GeneBank: J01415.2) 8500-8529	52
	Reverse (bio)	AATAAACCTAAAATTATAAAAACAATAAAT	(GeneBank: J01415.2) 8550-8579	
	Seq primer	AAATTATAATAAATTTTGAGAATTAAAATG	(GeneBank: J01415.2) 8500-8529	
	Seq entry	AAC/tGAAAATTTGTTC/tGTTTT	(GeneBank: J01415.2) 8530, 8549	
MT-TL1	Forward	TAGGGTTTGTTAAGATGGTAGAGTT	(GeneBank: J01415.2) 3222-3246	52
	Reverse (bio)	ACAATAAAAAATAAAAAATTAACCATAAAT	(GeneBank: J01415.2) 3309-3338	
	Seq primer	TAGGGTTTGTTAAGATGGTAGAGTT	(GeneBank: J01415.2) 3222-3246	
	Seq entry	C/tGGTAATC/tGTATAAAATTT	(GeneBank: J01415.2) 3247, 3254	
MT-ND5	Forward	GTGATATATAAATTTAGATTTAAATATTAA	(GeneBank: J01415.2) 12651-12680	52
	Reverse (bio)	TAAACAAAAAAAATATAATTCCTAC	(GeneBank: J01415.2) 12775-12799	
	Seq primer	TTAATTTTAGTTAT	(GeneBank: J01415.2) 12721-12734	
	Seq entry	C/tGTTAATAATTTATTTTAATTGTTTATC/tGG	(GeneBank: J01415.2) 12735, 12762	
CDH1	Forward	TTTGATTTTAGGTTTTAGTGAGT	Chr16: 68,770,918-68,770,940	55
	Reverse	ACCACAACCAATCAACAA	Chr16: 68,771,094-68,771,111	
CDKN2A	Forward	AGGGGTTGGTTGGTTATTAGAG	Chr9: 21,974,900-21,974,919	60
	Reverse	AAAACTCCATACTACTCCCC	Chr9: 21,974,792-21,974,810	
LINE-1	Forward	TTTTGAGTTAGGTGTGGGATATA	n/a	56.3
	Reverse	AAAATCAAAAAATTCCCTTTC	n/a	

Supplementary Information 2. Platelet mtDNA methylation level with age, BMI, and race

		Age			BMI			Race	
		P value	FDR adjusted P value		P value	FDR adjusted P value		P value	FDR adjusted P value
Healthy	MT-CO1	0.50	0.70		0.71	0.84		0.31	0.98
	MT-CO2	0.36	0.70		0.84	0.84		0.86	0.98
	MT-CO3	0.62	0.70		0.08	0.57		0.72	0.98
	MT-TL1	0.14	0.70		0.76	0.84		0.59	0.98
	MT-ATP6	0.70	0.70		0.67	0.84		0.98	0.98
	MT-ATP8	0.55	0.70		0.32	0.75		0.42	0.98
CVD	MT-CO1	0.77	0.77		0.07	0.23		0.42	0.69
	MT-CO2	0.21	0.58		0.37	0.65		0.32	0.69
	MT-CO3	0.59	0.75		0.05	0.23		0.20	0.68
	MT-TL1	0.33	0.58		0.95	0.95		0.74	0.86
	MT-ATP6	0.20	0.58		0.86	0.95		0.92	0.92
	MT-ATP8	0.65	0.75		0.73	0.95		0.17	0.68

A linear regression model was applied to compute associations between platelet mtDNA methylation with age, BMI, and race. Healthy individuals and CVD patients were handled separately due to differences in mean ages.

Supplementary Information 3. Scatter plots of mtDNA methylation from healthy individuals and CVD patients by age, BMI, and race

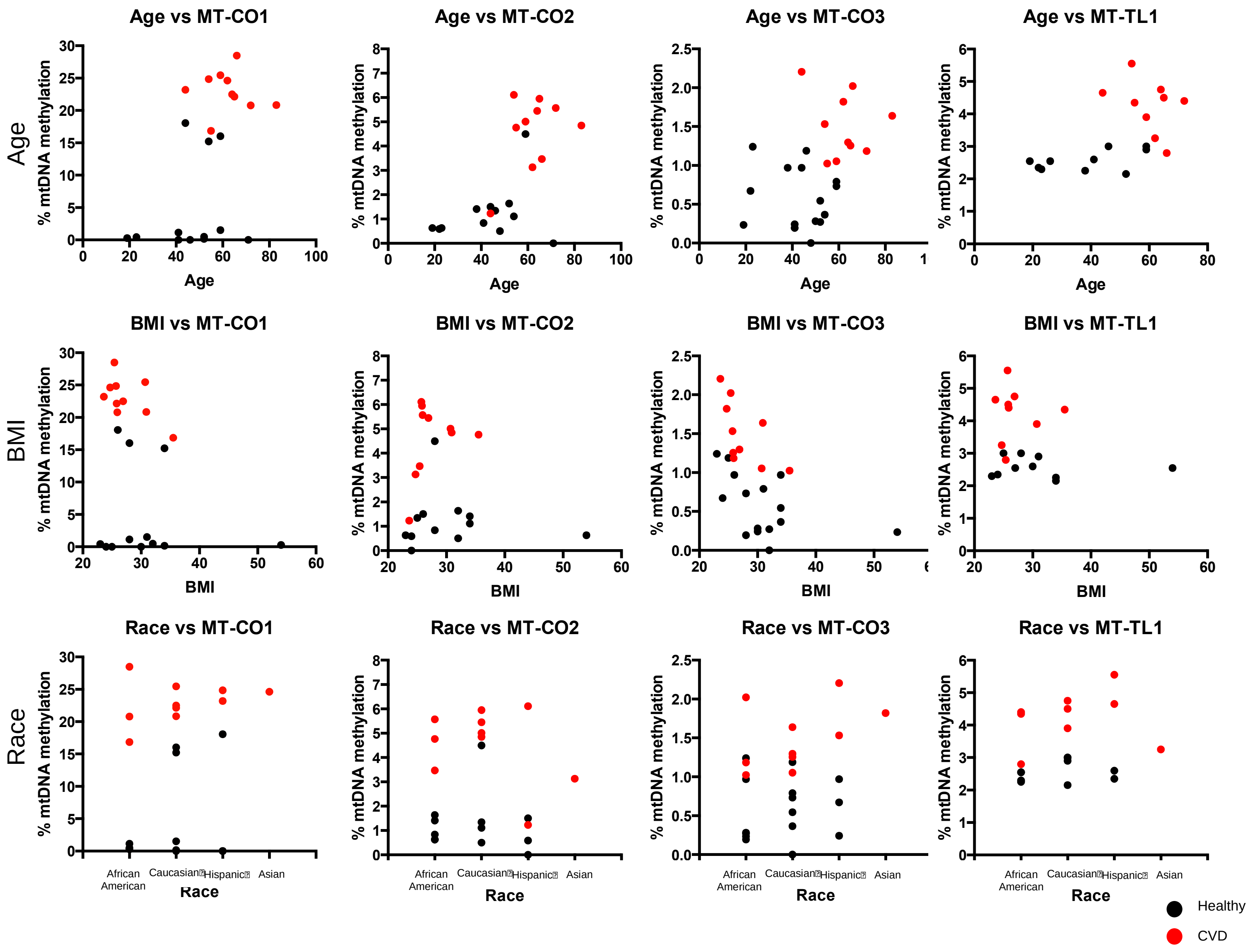

Supplement: Additional file 1: — Supplementary information 1, 2, and 3. Supplementary information 1. Primer sequence and information. Supplementary information 2. Platelet mtDNA methylation level with age, BMI, and race. Supplementary information 3. Scatter plots of mtDNA methylation from healthy individuals and CVD patients by age, BMI, and race. [file 13148_2015_78_MOESM1_ESM.pdf]
